# Turbulence in Nursing—A Conceptual and Contextual Exploration

**DOI:** 10.3390/nursrep16040119

**Published:** 2026-04-02

**Authors:** Helene Åvik Persson, Anders Palm, Karin Samuelson

**Affiliations:** 1 Department of Health Sciences, Faculty of Medicine, Lund University, SE-221 00 Lund, Sweden; anders.palm@litt.lu.se (A.P.); karin.samuelson@med.lu.se (K.S.); 2 Birgit Rausing Centre for Medical Humanities, Faculty of Medicine, Lund University, SE-221 00 Lund, Sweden

**Keywords:** turbulence, nursing care, concept analysis, contextual model

## Abstract

**Background/Objectives**: Contemporary healthcare systems are characterised by rapid change, high workload, and staff shortages, creating conditions that may compromise care quality and generate turbulence in nursing. Turbulence has been discussed in nursing research. However, greater conceptual clarity is needed regarding its underlying factors and implications for nursing work. The aim of this study was to explore and analyse the concept of turbulence in nursing and its related factors. **Methods**: The study was conducted using the Simultaneous Concept Analysis Method involving a consensus group and a nursing care, expert group and included content validity index ratings as a validation technique. **Results**: Eight factors related to turbulence in nursing were identified. A conceptual model was developed to illustrate the interrelationships among these factors and their role within the turbulence concept. Based on this model and the demonstrated contextual interconnections, a comprehensive definition of turbulence in nursing was formulated. **Conclusions**: The study has achieved a deeper understanding of the concept “turbulence in nursing” through the identification of eight different, generally valid turbulence-related factors and their presumed impact on nursing care. A conceptual model of interacting forces in turbulence in nursing has been presented as both a detector and a compass for mapping and counteracting future tendencies toward turbulence in the work environment. The study enables healthcare professionals and leaders to detect and address emerging turbulence in nursing practice and education. By clarifying its underlying major related sources, the model serves as a practical guide for improving the work environment, strengthening team resilience, and ultimately enhancing patient safety and quality of care.

## 1. Introduction

Healthcare systems globally are undergoing a period of significant change and strain [1,2]. Advances in medicine and technology have opened up new opportunities, while at the same time making the content and delivery of healthcare more complex [3,4]. An environment has emerged characterised by a high workload, staff shortages, rapid changes, and an increased need for healthcare professionals to be available [5], all contributing to growing pressure on healthcare systems [6]. Undesirable situations and circumstances can thus create a strenuous work atmosphere that in its entirety could be designated as turbulence, seriously affecting the quality of care and threatening long-term sustainability [7].

Managing turbulence arising from disruptions in fundamental nursing tasks has become a major issue in contemporary nursing care [7]. This is particularly evident in complex clinical environments, where registered nurses occupy a central position, balancing organisational demands from management with direct responsibility for patient care. Positioned at this intersection, nurses must continuously coordinate multiple tasks, roles, and expectations. This multifaceted role increases the risk of strain in situations characterised by high complexity, where competing demands may compromise person-centred, safe, and high-quality care. Such conditions may contribute to unreasonable workloads, stress related to feelings of inadequacy, and, ultimately, burnout [8,9,10,11,12].

At the same time, ongoing changes in healthcare practices, processes, and systems place increasing demands on the resilience in healthcare [13,14]. From the perspective of complexity science, healthcare systems can be understood as complex adaptive systems, in which organisational conditions and clinical processes interact dynamically. In such systems, even minor disruptions may have unpredictable consequences and contribute to instability and turbulence [15]. However, resilience in nursing is described as a dynamic process that involves adaptive responses to workplace adversity, and outcomes vary depending on the nature and manifestation of the challenges encountered [16]. Turbulence is a concept originally derived from physics to describe the complex and chaotic motions occurring in fluids, when the flow becomes irregular and difficult to predict. The origin of the term can be traced back to the writings of Leonardo da Vinci in the early sixteenth century, in which he described the swirling movements in water and air [17,18,19]. Turbulence is today a fundamental concept in fluid dynamics, used to describe unexpected movements in atmospheric winds as well as in the blood flow in our bodies [20]. A parallel could be drawn between unforeseen wind patterns and undesirable disturbances in healthcare environments, which strongly affect the conditions of the nursing profession. Convective currents in fluid or irregular jet streams in the air may be likened to the daily flow of patients, whereas extreme turbulence may arise on the verge of chaos, such as inadequate staffing levels in relation to patient acuity [21,22,23].

The metaphor of turbulence has been established as a scientific concept in an international context of nursing, and some research has been conducted to define and enhance the understanding of the concept. A study by Salyer [24] initially examined the effects of environmental turbulence on nurses’ work performance in an emergency hospital setting. Salyer’s study shows that the increasing number of patient admissions and discharges from a unit during a 24 h period had a negative effect on both performance and communication with patients, relatives and staff. Environmental turbulence has also been emphasised from the nurse’s perspective by Tillman et al. [25] in acute care settings and its impact on the ability to provide care. Nurses experience a lack of management of disruptive elements in the hospital environment, which manifests itself in a perceived loss of control over nursing care, increased workload due to reduced resources and staff, and a compulsion to take on tasks from other wards. Research has helped to clarify the contextual factors that may contribute to turbulence in the healthcare environment [26,27,28]. For example, Hawkins and Morse [27] highlight that nurses’ work is mainly influenced by contextual circumstances and that work tasks can get out of hand. A scoping review [26] describes how nurses use workarounds in an emergency department within healthcare and how these temporarily solved perceived barriers in the workflow, in order to manage the complexity of patient care. Recently, research has shifted towards understanding of what affects disruptions in workflow and situational awareness and, in turn, patient safety [7,29].

An early attempt to conceptualise turbulence in healthcare by Jennings [12] emerged when investigating contemporary conditions surrounding nurses’ work, such as communication and workload. Jennings [7] refined the contextualisation later to increase an understanding of turbulence, particularly by integrating it as a central factor in nurses’ workflows. An illustration of how communication (including interruptions and inadequate handovers) and workload (including high patient numbers and equipment issues) act as two primary sources of turbulence, is a prominent feature in the study. Several studies have hereafter been conducted that include workload [30,31,32,33]. Moreover, studies have been performed to explore how specific circumstances, such as patient turnover, issues with materials and equipment, staffing resources, and the physical environment contribute to turbulence [27,32,34,35]. The understanding of the complexities in nursing practice by applying the concept of turbulence may support efforts to define and describe key factors relevant to nurses’ work environment and job conditions.

Previous research has explored turbulence within nursing from an international perspective, mostly originating from the United States, shedding light on its determinants, and consequences in healthcare practice [7,12,30]. However, there is a need to further identify and discern interactive factors that are related to the development of turbulence. If turbulence in nursing is better understood and managed, the conditions for delivering correct, safe and optimal care will improve, and thereby so will the patients’ health and wellbeing. The aim of this study was therefore to explore and analyse the concept of turbulence in nursing and its related factors.

## 2. Methods

### 2.1. Setting

The immediate prerequisites for the exploration of the key concept and its contexts are based on our special knowledge, studies and experiences of nursing practice within the Swedish healthcare system. The system in Sweden, contains shared responsibility between the government, 21 regional health authorities, and 290 municipalities. The care that is needed is made universally accessible by the system and is mainly tax-funded, although there are some private providers [36,37]. The goal of Swedish healthcare is to ensure good health and equal care for all, in which human equality and individual dignity is respected, with priority given to those in greatest need [38]. The Swedish healthcare system is divided into primary healthcare, which includes nursing homes, home care services and primary healthcare centers, emergency care, including pre-hospital paramedic care and hospital-based care and inpatient/specialised healthcare, which refers to hospitals [37,38]. Registered nurses are the largest category of staff in Swedish healthcare with a university education in nursing sciences at bachelor’s level or above, and approximately 4 of 10 nurses have a specialist education. The specialist nursing programme in primary health care accounts for the largest share, followed by anesthesia and intensive care [39]. These specialist nursing programs lead to a one-year degree of Master of Medical Science [40]. The core competencies of registered nurses in Sweden include person-centred care, safe care, teamwork, evidence-based care, improvement knowledge for quality development and informatics. The latter involves the planning, implementation and evaluation of nursing care. Furthermore, their skills and responsibility also include participating in and, when necessary, conducting assessments and treatments. In addition to their direct patient care responsibilities, registered nurses are also responsible for the supervision and training of other healthcare professionals. Collaboration with patients, next of kin, other professionals and healthcare providers, is also a part of their work [11].

### 2.2. Concept Analysis

This study involves a contextual exploration of the concept of turbulence in nursing, using a concept analysis, where the concept is specified in its interaction with familiar phenomena in the nursing practice [41]. Contextual exploration may comprise various methods used to derive the main conceptual meaning, for example, through various descriptions of the contextual characteristics and practical implications of the concept [42]. The Simultaneous Concept Analysis (SCA) method, which was introduced by Haase et al. [43,44] and originated from Rodgers’ evolutionary perspective [45], has inspired this study. The aim of the method is to analyse the interrelationships of individual concepts and to define characteristics, outcomes and themes by identifying and comparing different connections. Furthermore, it aims to clarify theoretical, empirical and practical confusion between concepts and to deepen understanding of the processes behind each phenomenon [44,46]. This process allows for clarification and theory construction that is relevant to nursing and SCA is an essential part of the development of knowledge, scientific discipline and professional language [44]. The strategy used in this study contained several steps in accordance with SCA, which has been used flexibly and iteratively including a consensus group process, as well as a content validity index calculation.

#### 2.2.1. Developing a Consensus Group

The diversity of perspectives in the consensus group is one of the main requirements of applying this method [44]. The consensus group consisted of three members (AP, KS, HÅP), all of whom were researchers with individual expertise within different research areas. Our first member (AP), senior professor in comparative literature and medical humanities, working with interdisciplinary concepts and their interrelationships, served as group moderator. The second member (KS) is specialised in nursing and intensive care with an expertise in quantitative methodology and assumed the role of consistently prompting consideration of how the results of the SCA could influence the practical evaluation of the interrelations between the concepts. The third member (HÅP) also specialised in nursing, with a focus on public healthcare, served as the qualitative expert. The consensus group met once a month over a ten-month period.

#### 2.2.2. Selecting the Concept to Be Analysed

The consensus group initially held a meeting where they, driven by their concordant research interests, developed strategies for constructing theoretical descriptions of the key concept of turbulence. The focus on turbulence and turbulence-related factors was based on their clinical relevance within various nursing settings, together with the lack of recent analytical studies from a Swedish perspective addressing this concept both in its breadth and depth and applying a different methodological approach.

#### 2.2.3. Refining the Concept Clarification Approach

The next step in the analysis for the consensus group was to determine which concept clarification technique to apply. The use of a nursing care expert group was considered the best approach. Registered nurses employed at the department of Health Sciences at Lund University were therefore informed about the study and asked to participate. After formal approval, the group consisted of 16 members with clinical experience in nursing including anaesthesia, intensive care, paediatrics, public health nursing, oncology and radiography nursing. This expert group had an extensive expertise of various deliveries of services within healthcare, consisting of healthcare professionals and researchers who work or have worked in the Swedish healthcare system.

#### 2.2.4. Clarifying and Revision of the Individual Concepts

To clarify the individual, interactive concepts, i.e., turbulence-related factors, each researcher in the consensus group worked independently to explore what was encompassed within different factors, such as sources and causes related to turbulence. First, preliminary definitions, antecedents, essential attributes, and potential outcomes emerged through this independent process. This resulted in eight preliminary turbulence-related factors that were outlined as the concepts of interest: competence differences, lack of continuity, weak leadership, insufficient resources, workload, technical problems, cooperation problems and deviations from routine. This was followed by a workshop involving the expert group, which began with an introductory presentation of the key concept and the rationale behind the selection of factors to be analysed in their relation to turbulence.

The participants then worked in pairs to review the drafts developed by the consensus group, applying the eight suggested turbulence-related factors within the context of their own knowledge, their clinical experience, and examples drawn from the healthcare sector across each proposed turbulence-related factor. A plenary discussion then took place, where the various turbulence-related factors were explored in greater depth, and any uncertainties or ambiguities were addressed, particularly regarding what should be included within each concept. Hereafter each pair documented their reflections on the eight factors including examples from their own clinical experience.

The consensus group then met to analyse and discuss the expert group’s feedback on the eight preliminary concepts. The consensus group met on several occasions to revise the concepts based on the feedback from the expert group. This process continued until everyone in the consensus group reached agreement on each concept. One major revision was a change in terminology of the included concepts, from merely presenting causes of turbulence to the sources. For example, “insufficient resources” was renamed “resources” and thus each individual concept became neutral, open for and receptive to different specifications.

#### 2.2.5. Developing and Re-Examining the Validation Matrices

As a validation technique, within the scope of the SCA method, we chose to perform a content validation using the content validity index (CVI) calculation to ensure that the selected factors adequately represented the full scope of the key concept of turbulence. This step was undertaken to validate the conceptual exploration with empirical evidence derived from expert evaluation, in line with the recommendations of Polit and Beck [47]. Content validity is often established through the calculation of a CVI, which is derived from item-related ratings provided by subject matter experts [48]. Thirteen individuals in the nursing care expert group rated the relevance of eight turbulence-related items on three different occasions in this study. The first CVI ratings were taken at the end of the workshop, and the two following ratings were carried out online. The consensus group identified some minor inconsistencies and subsequently revised the items based on the first and second CVI results. The final overall CVI rating was satisfactory (recommended limit > 0.8) [48], yielding an average CVI of 0.94 for all the items (Table 1). Despite the fact that one item, “technology” had a lower CVI of 0.77, the consensus group decided to retain the item, due to previous higher CVI results and our own experience of technology in healthcare such as breakdowns and malfunction.

#### 2.2.6. Developing a Conceptual and Contextual Model

**Figure 1:**
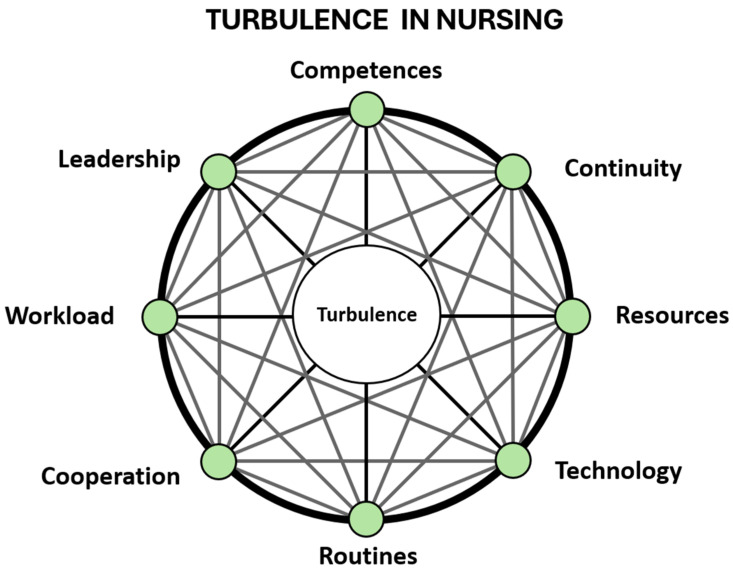
Model of Turbulence in Nursing with its eight inter-related factors. The model is designed as a holistic contextual illustration in shape of a circle, focusing the key concept turbulence as its centre and the corresponding contextual factors as points on the circumference. Diameters, radii and chords (lines connecting any two points on the circumference) create the elaborate network of possible interrelations (for an animated, stepwise PowerPoint version of the model illustrating the interactions between factors, see Supplementary Materials).

One of the members (AP) in the consensus group developed a conceptual and contextual model for examination and explanation of the relationships between the key concept, turbulence, and its selected eight interrelated factors (Figure 1). The identification of the eight key factors in the clinical environment and their interactive relation to turbulence constitute the basis for our model, visualizing the complex contextual network of interdependencies in which turbulence in nursing had to be understood. The interrelated factors are all named as multidimensional entities, each demanding exemplification and specification to be intersubjectively validated, and adapted to the individual specific environment and situation.

The function of the model could be described as a detector and a compass. Detector—meaning that the model helps us to discover the complicated mixed interactions between turbulence and the sources as well as mutually between the interconnected sources. Compass—meaning that the model helps us to uncover the specific directions in which to search for the prominent problems in the individual situation. The model presents a visual, broader and more distinct, view of turbulence in nursing than most of the proposals in scientific studies so far. Accordingly, our model corresponds to a standard definition formulated as follows. A model may be understood as a simplified and interpretable representation of a phenomenon, designed to capture its essential properties and relationships [49]. In conclusion, the model provides us with guidelines to our consistent definition of turbulence in nursing.

#### 2.2.7. Submitting the Results to the Experts for Criticism

The results of the SCA and CVI were presented to the expert group during a seminar who were given the opportunity to discuss and review the manuscript. Consensus was reached after final revisions.

## 3. Results

### 3.1. Description of Turbulence-Related Factors

We aimed to create a stipulative wide-ranging definition of turbulence and have identified eight turbulence-related factors that are significant for the outcome of turbulence: competences, continuity, cooperation, leadership, resources, routines, technology and workload (Figure 1). There is an obvious risk in the nurse’s working environment of deviations in the workflow such as disruptions, obstacles, interruptions, incidents and distractions. If these deviations become too numerous, recurring or mutually interactive, they can cause turbulence in the nurses’ workflow. Turbulence may begin in one turbulence-related factor, triggering ripple effects that impact others sequentially. These deviations could accumulate over time potentially and at worst escalate into a loss of control or even chaos within the system. However, it should be noted, a certain amount of turbulence may have a positive impact on workflow, as it may encourage nurses, and staff to streamline their working methods and promote a more solutions-oriented approach in the workplace.

#### 3.1.1. Competences

Competence in nursing means the ability to perform the duties of one’s profession in general or a specific professional task with a satisfactory level of skill, training and education. Competence in nursing refers to individual qualifications such as education, nursing care and life experience, as well as team and interdisciplinary competence. One example of when competence could lead to turbulence in nursing may appear when a patient with acute sepsis needs to have a peripheral venous catheter inserted. The nurse lacks sufficient competence to deal with difficult vascular access and fails several times, which delays medication treatment. The delay leads to a deterioration in the patient’s condition, an emergency alarm, and several staff members having to interrupt their ongoing work to stabilise the patient.

#### 3.1.2. Continuity

Continuity in nursing means coherent events over time and implies an uninterrupted and consistent sequence, but not one that is unchanging. Continuity in nursing refers to consistency in tasks, staff turnover, leadership and organisation. It is also about being able to carry out one’s duties without being interrupted frequently. One example of how continuity can lead to turbulence in nursing is when the nurse in charge does not follow up on important information about a patient’s condition. This may occur when the nurse in charge is not caring for the same patient throughout the entire care period, for instance due to scheduling issues or unexpected interruptions in continuity. This could lead to important information about the patient’s condition being lost, a decline in the quality of care, and an emergency arising that disrupts the work in the unit.

#### 3.1.3. Cooperation

Cooperation in nursing means a joint effort at different levels to achieve a common goal and can take place within and between, for example, healthcare organisations, government agencies and stakeholders, and may involve the active exchange of information and values or the planning of joint activities. Cooperation in nursing is enabled by communication within and across professional groups, between management and staff, and between staff and patients. It can promote dialogue for shared learning and decision making to achieve high quality and safe care. One example of how collaboration could lead to turbulence in nursing care is when there is a lack of collaboration due to poor communication. This may result in important information about the patient’s general condition not being shared in time, which delays action and disrupts the work of the care team.

#### 3.1.4. Leadership

Leadership in nursing means an ability to direct and influence the actions and attitudes of an individual or group. It is about setting goals and providing guidance, communication and support. Leadership in nursing involves responsibility and delegation, while ensuring that supportive structures are in place, while also including responsibility for crisis management, preparedness, quality assurance and patient safety. One example of leadership in nursing that could create turbulence is poor crisis management. If, in the case of unexpected events, there is a lack of leadership in the form of swift action and clear directives, confusion may arise and coordination among staff could deteriorate. This could lead to chaos.

#### 3.1.5. Resources

Resources in nursing are necessary for the provision of health and medical care and include human resources, healthcare facilities, financial resources, equipment and materials used in the provision of healthcare services. Resources in nursing also includes funding and administrative resources and support functions related to the provision of healthcare services. One example of when turbulence in nursing care could arise in relation to resources is when staffing levels are lower than planned. This forces nurses to reprioritise their tasks, increasing the risk of delayed care and reduced patient safety and wellbeing.

#### 3.1.6. Routines

Routine in nursing means a predetermined approach to how a task should be performed, and it includes responsibility for the activity. A routine also includes what should be done, how it should be done and how often it should be done. Routines in nursing can consist of governing documents, care procedures, schedules, task distribution and reporting. One example of when turbulence in nursing care could arise in relation to routines is when a patient is transferred between two wards and there is no routine for transferring information and reporting. This creates a risk that important patient information will be communicated incompletely or in the wrong order.

#### 3.1.7. Technology

Technology in nursing refers to the use of information technology to support the management, administration and processing of medical and nursing data, using tools such as computers or medical devices in healthcare. Technology supports practical care to best meet digital needs and includes the collection, storage, presentation, transfer and communication of data and information. One example of how technology could create turbulence in nursing refers to a historical example, the millennium crash, where a system error in the medical record system prevented nurses from documenting patient information correctly. This led to delays in care, patient safety risks, increased workload and stress in the department.

#### 3.1.8. Workload

Workload in nursing means a measure and quantification of the total amount and complexity of care needed at a given time. A high workload in nursing can arise even with a relatively low number of patients, if the complexity of the patient’s care need is extensive and time consuming. One example of when the workload could lead to turbulence in nursing is when there are several patients in the ward who have multiple diseases and require advanced medical treatment and nursing care, despite there being no overcrowding of patients. These patients therefore require significantly more time than the average for patients, and the available staffing is insufficient to meet all the needs, leading to prioritisation dilemmas.

#### 3.1.9. Definition

The following definition, launched by the consensus group, is based on the conceptual and contextual model accompanied by the above explanations of the eight turbulence-related factors.

*Turbulence is a process or a state that may arise when disruptive deviations in the workflow of nursing care occur and interact within the frameworks of one or more of the following eight principal factors in the clinical environment: competences, continuity, cooperation, leadership, resources, routines, technology and workload.*

## 4. Discussion

The major finding in the study is the development of the definition of turbulence in nursing, derived from our model consisting of eight turbulence-related factors, which offers an instructive and cohesive understanding of the concept not previously presented in this form in international research [7,30]. By integrating a combinatorial methodology with a new contextual model, the study contributes to a broader global understanding of how interacting factors shape the nursing work environment, which, to our knowledge, has not been conducted in the same manner before. Although grounded in the Swedish healthcare context, with its distinctive combination of universal access, advanced specialization, and staffing challenges, the findings have clear international relevance. The model provides a theoretical lens for visualizing and analyzing the complexity of nursing practice, highlighting the balance required among the eight factors to maintain workflow stability and prevent disruption.

The use of turbulence as a metaphor for the conditions of the nursing profession merits consideration. Nurses often occupy the role most exposed to the pressures of turbulence, with competing responsibilities, fragmented workflows, and constant demands for prioritization. It could be argued that nurses function as the hub in the clinical environment. Turbulence can manifest as nurses’ multitasking, time pressure, and ethical strain, contributing to chronic stress, exhaustion, and burnout [8,50,51,52]. When turbulence undermines the ability to provide safe and high-quality care [11], it may also create organizational overload and “change fatigue” [53]. These effects extend beyond the individual nurse, influencing teamwork, the work climate, and ultimately the quality and efficiency of patient care. As extensively explored in previous research [31,54], workload remains central but insufficient on its own to capture the complexity of nursing care demands. This is despite the fact that it has been exhaustively defined in different ways, and several instruments have been developed [33,55,56,57,58,59,60,61]. It should be noted in our model that workload is integrated as one of several interacting factors that shape disruptive deviations in nursing workflow.

The Swedish healthcare system, despite its strengths in access, quality, and digitalization [62], faces challenges common across many countries: lack of continuity, coordination difficulties, and staff shortages [36]. Swedish legislation [63] and the national competency description for registered nurses [11] emphasize safe and high-quality care, yet turbulence arises when even minor disruptions trigger chain reactions that destabilize the system. As a result, stress and disruption arise in a healthcare system designed to maintain security and continuity [37]. This aligns with current research showing how inadequate work environments increase burnout, cognitive load, workarounds, and risks to patient safety [64]. Healthcare organizations also face inherent complexity, making unpredictability an expected characteristic of everyday work.

Although this study is grounded in the Swedish healthcare system, the turbulence-related factors identified reflect fundamental dimensions of nursing practice that are present across healthcare system globally. While organizational structures, resources, and regulatory frameworks may differ between contexts, the underlying conditions of clinical work, such as coordination, workload, competence, and access to resources, are broadly shared. Differences between healthcare systems are therefore more likely to influence the relative prominence and interaction of these factors rather than their existence. In this sense, the model is intended as a context-sensitive but transferable framework for understanding and analyzing turbulence in practice.

There are a number of strategies that are designed to improve the organization of healthcare. Firstly, Spears [65] points out how successful organizations integrate continuous improvement into routine practice and actively engage staff in change processes, a so-called employee-driven continuous improvement, principles that are highly relevant for nursing. Furthermore, another aspect that Spears has highlighted is that successful organizations strive to improve their workflows [65]. Registered nurses can focus more on patients’ needs when the workflow proceeds without unplanned disruptions, thereby reducing the risk of certain care measures being neglected. This phenomenon is described in the literature as missed nursing care [66,67]. Previous research has shown that missed nursing care is clearly associated with negative patient outcomes, including increased mortality and longer hospital admissions [66,67,68]. An improved workflow in nursing can increase value for patients and staff, creating person-centred care. Functional workflow should therefore be regarded as a key component of patient safety.

Furthermore, recruitment and retention of registered nurses remain pressing international challenges. Decisions to leave the profession are multifaceted and influenced by psychosocial work environment factors such as staffing levels, workload, leadership, and salary [69,70]. Initiatives such as Magnet4Europe [71] aim to create work environments that promote staff empowerment and professional development, thereby strengthening retention [72]. These organizational characteristics overlap with several of the turbulence-related factors in our model, underscoring its potential relevance for workplace development and policy.

The contextual turbulence model presented in this study serves as both an analytical and diagnostic tool, supporting a clearer understanding of how turbulence emerges and the implications it may have for nursing practice. To illustrate how the model may be applied, a deficiency in competence in a specific clinical situation may lead to delays in care, which in turn increase workload for other staff and disrupt continuity and cooperation within the team. Conversely, limited resources may intensify workload and reduce time for communication, thereby affecting both cooperation and patient safety. In this sense, the model functions as a “detector” by helping to identify interacting sources of disruption, and as a “compass” by indicating where to focus attention in order to address emerging turbulence in a given context. These examples are illustrative and context-dependent, as the strength and direction of interactions between factors may vary across clinical situations.

The practical usefulness, its application power, may be particularly relevant in nursing education, where it can be used to illustrate system complexity and support reflective learning. The definition and model also point towards the need for further research exploring turbulence from different perspectives. The ongoing development of the Turbulence Rating Assessment Checklist (TRAC), abbreviated Lu TRAC, illustrates one such direction, aiming to identify turbulence-related factors at an early stage in somatic wards to prevent escalation. Future studies may further investigate the consequences of turbulence through in-depth qualitative work, enabling a richer understanding of how staff experience turbulence and which factors are perceived as most influential across different settings.

### Strengths and Limitations

A key strength of this study lies in its systematic and theoretically grounded conceptual approach, which enabled a deeper understanding of the multifactorial phenomenon of turbulence in nursing [44]. The development of a contextual model integrating eight turbulence-related factors provides a comprehensive framework that clarifies the complex interrelations shaping turbulence in nursing care. Analytical rigor was strengthened through the combined use of a consensus group and an expert group, ensuring multidisciplinary perspectives and intersubjective validation [44,73]. In addition, CVI [48] was also applied as a validation technique, which increased the credibility of the study [73]. Furthermore, the model’s flexible structure, allowing each factor to be interpreted and adapted to different clinical environments, enhances its potential relevance for both practice and research. Using the model as a blueprint, a new well-founded definition of turbulence in nursing has been introduced.

The model and the subsequent definition have significant utility value, both as a foundation for future empirical research and as a pedagogical resource in nursing education at various levels. In practice, the model may support leadership decision-making by facilitating the identification of interacting sources of disruption in clinical workflows. It may also inform workforce planning by highlighting imbalances between key factors such as workload, resources, and continuity. In educational contexts, the model can be used to support reflective learning and to illustrate the complexity of nursing practice.

This study also has certain limitations. The conceptual model is based primarily on theoretical analysis and expert interpretation rather than empirical testing, and its applicability requires further examination in diverse healthcare settings. The fact that the model has not been empirically tested can also be seen as a natural part of an iterative research process, where theoretical development precedes empirical testing [43,44,45]. The decision to retain the turbulence-related factor technology despite the lower CVI value of 0.77 might be a limitation of the study. This result may reflect the fact that technology has a major impact on several nursing care domains but less in others. Moreover, although the expert panel represented several nursing care domains, the data were drawn from one geographical region within the Swedish healthcare context, which may influence transferability to other contexts [73]. As the understanding and manifestation of turbulence can vary across organizational and cultural settings, further empirical refinement and validation are warranted. However, the consensus-based and conceptual nature of the study is consistent with its aim to develop a context-sensitive and theoretically grounded understanding of turbulence in nursing. Despite these limitations, the study provides a novel theoretical contribution that lays a robust foundation for future empirical investigation and subsequent practical application.

## 5. Conclusions

Based on a newly developed contextual model, our aim was to achieve an overarching and more comprehensive understanding of the phenomenon of turbulence in nursing. Our study calls attention to turbulence as a central concept for understanding the conditions of contemporary nursing practice. Turbulence arises from the inherent complexity and unpredictability of healthcare systems, where even minor disruptions can provoke chain reactions that influence workflows, teamwork, and the quality, safety, and efficiency of care delivery.

Recognizing and addressing turbulence is therefore critical. Using the definition and the eight identified turbulence-related factors as an analytical framework, healthcare organizations can better identify sources of disruption, support nurses in managing them, and develop strategies to mitigate their negative impact. Such efforts are essential both for sustaining the nursing workforce and for securing safe and effective patient care. Our conceptual and contextual exploration has been synthesized into an original circular model functioning both as a detector and as a compass, guiding us toward a new comprehensive definition of turbulence in nursing.

Taken together, the model and definition provide a contextually grounded platform for future empirical examination of turbulence in diverse clinical environments, while also offering an immediate framework that can be integrated into nursing teaching to support deeper understanding of the complexity in practice.

## Figures and Tables

**Table 1:** Content validity index of items related to Turbulence in Nursing. The degree of relevance for each item was judged by 13 nursing care experts.

Items	Experts in Agreement, *n*	I-CVI
Competence	12	0.92
Continuity	13	1.00
Cooperation	13	1.00
Leadership	13	1.00
Resources	12	0.92
Routines	12	0.92
Technology	10	0.77
Workload	13	1.00
S-CVI		0.94

Experts in agreement; number of experts giving the item a relevance rating of 3 or 4 (1 = not relevant, 2 = somewhat relevant, 3 = quite relevant, 4 = highly relevant). I-CVI = item-level content validity index (the proportion of content experts giving item a relevance rating of 3 or 4). S-CVI = scale-level content validity index (the average).

## Data Availability

The datasets analysed during the current study are available from the corresponding author on reasonable request.

## Supplementary Materials

The following supporting information can be downloaded at: https://www.mdpi.com/article/10.3390/nursrep16040119/s1, Figure S1: Turbulence in Nursing.

## Public Involvement Statement

There was no public involvement in any aspect of this research.

## Guidelines and Standards Statement

This manuscript was drafted using the recommended steps in the description provided for Simultaneous Concept Analysis method [43,44].

## Abbreviations

The following abbreviations are used in this manuscript:

CVI	Content validity index
SCA	Simultaneous concept analysis
