# Supplementary material for: Turbulence in Nursing—A Conceptual and Contextual Exploration
Source: Nurs Rep. 2026 Apr 2;16(4):119. doi: 10.3390/nursrep16040119 (PMC13118475; doi:10.3390/nursrep16040119)
Supplement: Supplementary file 1 [file nursrep-16-00119-s001.zip › nursrep-4190351-Supplementary.pptx]

## Slide 1
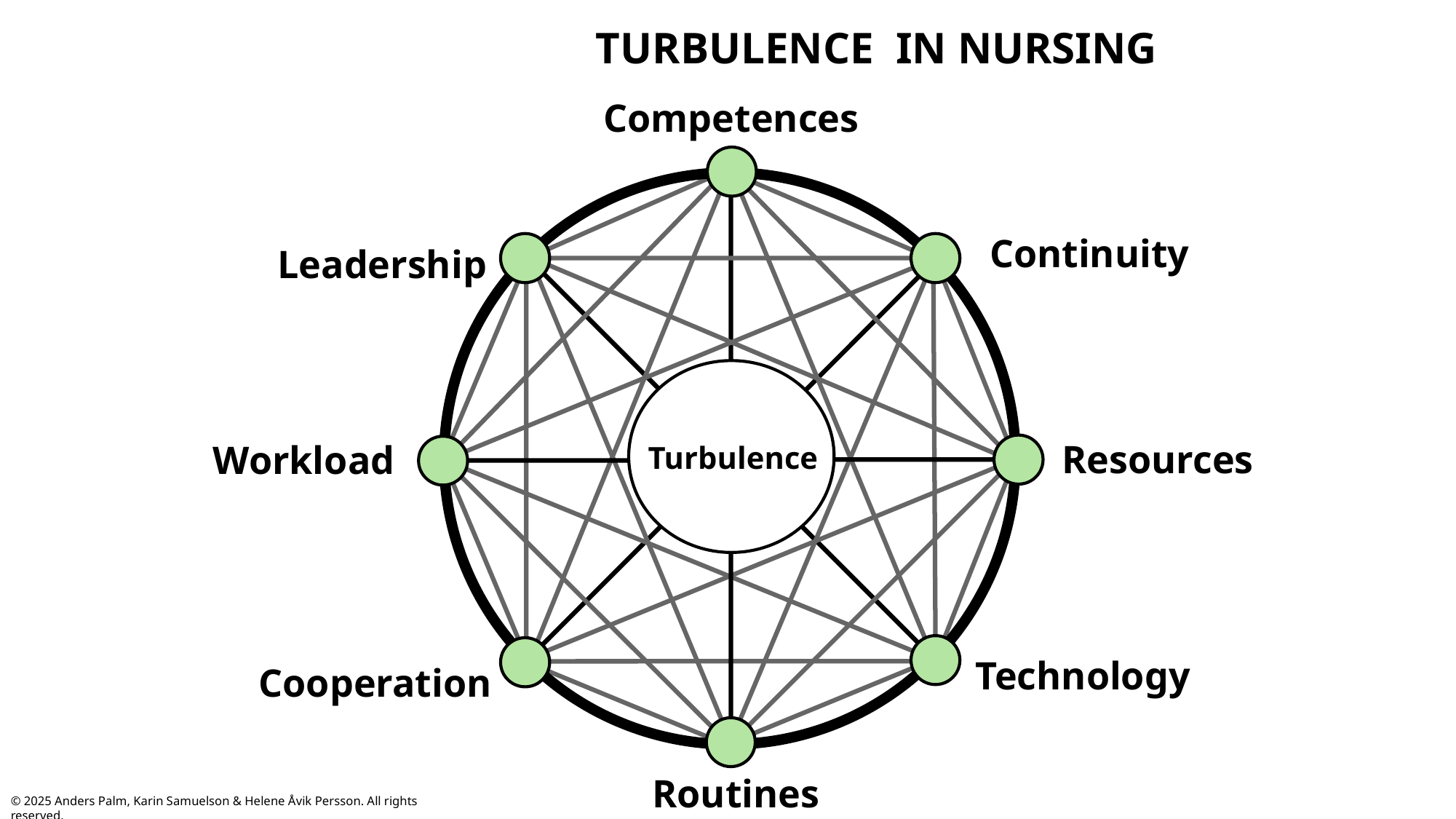

# TURBULENCE IN NURSING
Competences
Continuity
 Leadership
Resources
Workload
 Turbulence
Technology
Cooperation
 Routines
© 2025 Anders Palm, Karin Samuelson & Helene Åvik Persson. All rights reserved.
